# The use of joints of meat as phantoms for ultrasound-guided needling skills: a prospective blinded study

**DOI:** 10.1186/s13089-022-00263-9

**Published:** 2022-03-26

**Authors:** Jasmine Samuel, Euan Kerr, David Young, Malcolm Watson, Diana Raj

**Affiliations:** 1 Department of Anaesthesia, Queen Elizabeth University Hospital, 1345 Govan Road, Glasgow, G51 4TF Scotland; 2 Department of Mathematics and Statistics, University of Strathclyde, Glasgow, Scotland

**Keywords:** Education, Ultrasound phantom, Ultrasound-guided regional anaesthesia training

## Abstract

**Background:**

Needle visualisation during ultrasound-guided procedures is a skill that can be difficult to practise, with commercially available phantoms being expensive and often unrealistic. Our aim was to find an inexpensive, reproducible model that could be used to assist in developing this skill.

**Methods:**

Pork shoulder, beef brisket, and lamb shoulder joints were compared to a standard blue ultrasound phantom. Five ‘chunky’ yarn pieces were twisted together and threaded through each joint to simulate hyperechoic nerves. Participants were instructed to ultrasound each specimen and insert a needle close to a nerve like structure. Using a visual analogue scale, specimens were scored based on realism of appearances of ultrasound images and ‘feel’ of needling.

**Results:**

38 people participated. All specimens of meat scored significantly higher than the blue phantom (*p* = 0.01). There was no significant difference between the different types of meat.

**Conclusions:**

Pork, beef and lamb joints are an effective model to use for simulation training for needling skills. They have limited lifespan, but due to its relatively low cost, it is feasible to discard the meat after each training workshop. We hope the use of inexpensive meat products will make ultrasound simulation training simpler to organise and more effective.

## Background

Ultrasound-guided procedures are common practice in many medical specialities and skills in this area are required by many professionals in the health service. Independent performance of ultrasound-guided regional anaesthesia (UGRA) is a compulsory part of the anaesthetic curriculum in the UK [1]. It forms a vital part of perioperative pain management and leads to improved patient satisfaction [2–4]. Ultrasound guidance allows real-time visualisation of anatomy, and avoidance of critical structures [5].

Proficiency in the art of UGRA involves three fundamental parts: the understanding of basic physics of ultrasound, recognition of sonoanatomy and the mastery of needle visualisation. While the first two parts can be safely learned using textbooks, multimedia platforms, and scanning of models; the mastery of needle visualisation can be challenging [6]. It requires fine motor skills and transducer–needle coordination to ensure visualisation of the needle tip. *Gibbs* showed that medical training using simulators specifically for ultrasound training had been effective, and practice through simulation can reduce complications and increase confidence [7–9].

Simulators can vary in sophistication from low-cost part-task trainer phantoms to high-fidelity complex computer-based machines. The latter have a significant cost [10]. Designs of in vitro models have ranged from water baths, gelatin or rubber based, animal based, realistic cadavers, and computer-based virtual reality models. Homemade gelatin or agar preparations are cheap and accessible, but can be labour intensive to make, unrealistic, and artefacts can form after repeated use. Commercial phantoms are durable and easy to store, but are relatively expensive, consisting of a homogenous structure with a low background echogenicity [11]. Cadavers provide a very realistic model with good tactile feedback and correct anatomical relationships, but are inaccessible, expensive, and require specialist storage. Ideal benchtop part-task trainer models would be portable and easily accessible.

Research has shown that animal meat models provide a comparable experience to cadaver models for training in regional anaesthesia, and multiple meat-based models have previously been described [12–16]. Pork has been used in many studies, but, to our knowledge, there has been no description of the use of beef or lamb as an ultrasound phantom model. Our aim was to compare the realism of the different types of meats, specifically to assess the realism of the appearance of ultrasonic images obtained and the ‘feel’ of needling in comparison to human subjects.

## Methods

This prospective blinded study was carried out in June 2021, over two days, at an UGRA training workshop in the West of Scotland. Ethics approval was not required as there were no human subjects recruited to this study. All participation was voluntary.

At the workshops, ultrasound sonoanatomy of various peripheral blocks were demonstrated on live models. The needling station taught hand–eye coordination and the mastery of needle visualisation. Specimens labelled A to D were displayed at this station. Candidates and faculty were invited to take part by assessing the realism of the ultrasound image and the needling process of the specimens compared with their prior experience performing blocks on real patients.

**Fig. 1:**
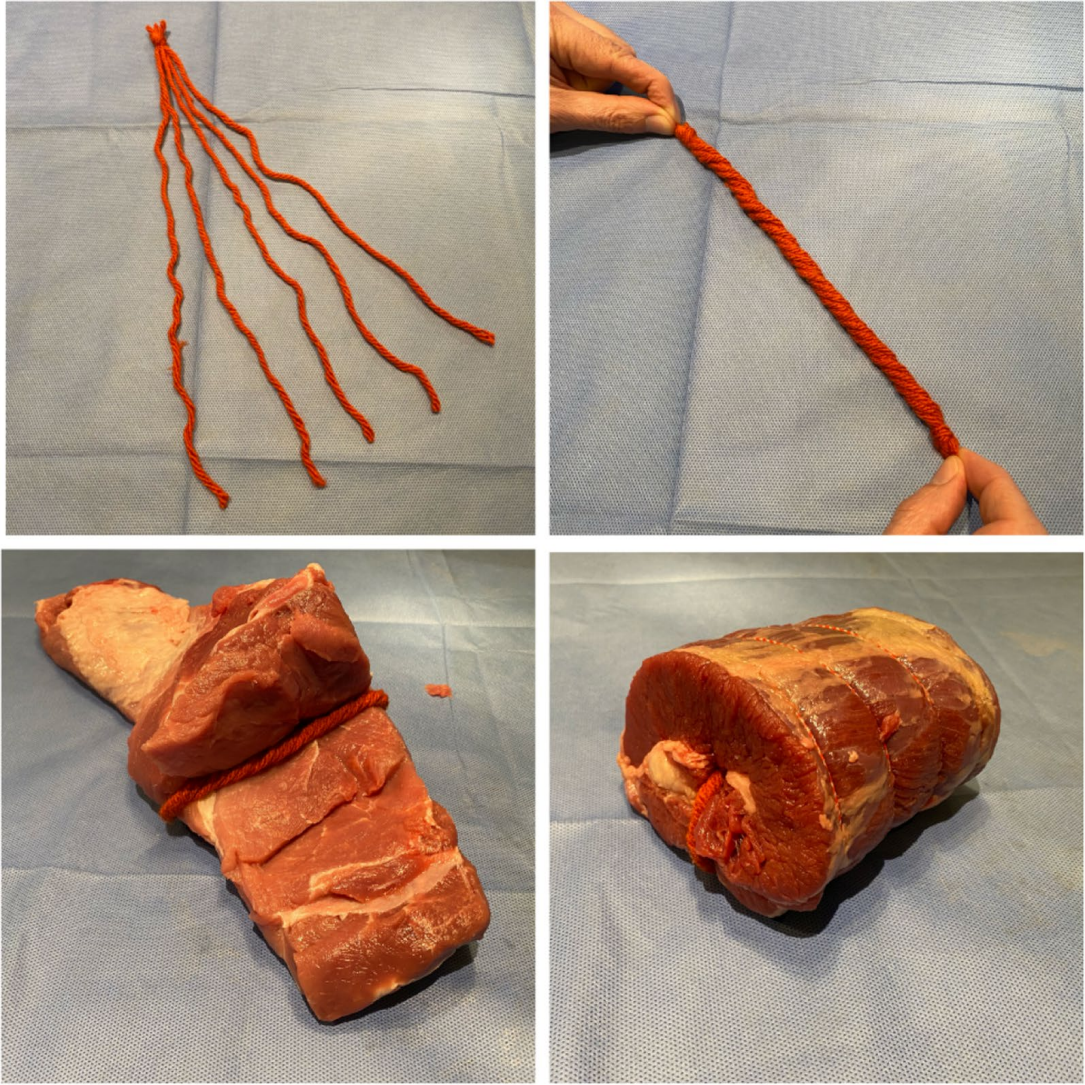
Preparing the meat

The meats used for specimens A, B and C were pork shoulder, beef brisket and lamb shoulder, respectively. Each meat weighed between 0.9 and 1.5 kg. Five pieces of ‘chunky’ yarn were soaked in water, twisted together and inserted into the natural fascial layers of the meats and secured using a handheld suture (to stop the twists unravelling). The yarns were laid approximately 5–7 cm from the top of the pieces of meat to simulate realistic, hyperechoic peripheral nerve structures [17] (see Fig. 1). External packaging and thick skin (particularly around the pork shoulder) was removed from the meat before it was wrapped in a single layer of cling film and anonymously labelled as specimens A to C. Participants were blinded to the type of meat used as specimens.

A standard blue phantom (Blue Phantom™ Ultrasound Training Model Select Series for Peripheral Nerve Block) was labelled specimen D. The Blue Phantom is made of elastomeric rubber that has the capacity to self-heal with repeated needling. This is due to the rubber having an affinity for itself which reconstitutes on withdrawal of the needle. This is used widely as a phantom model for training in UGRA.

All participants were asked to perform an ultrasound scan of each specimen and insert an ultrasound compatible echogenic needle (Stimuplex® Ultra 360° 20G 100 mm B Braun, Melsungen, Germany) simulating the needling process of UGRA. These needles have a 360° ‘X-pattern’ on the distal 20 mm for better visualisation and a 30° back-cut bevel for consistent puncture. Each participant had approximately 15 min to complete their assessment of realism for all four specimens.

**Fig. 2:**
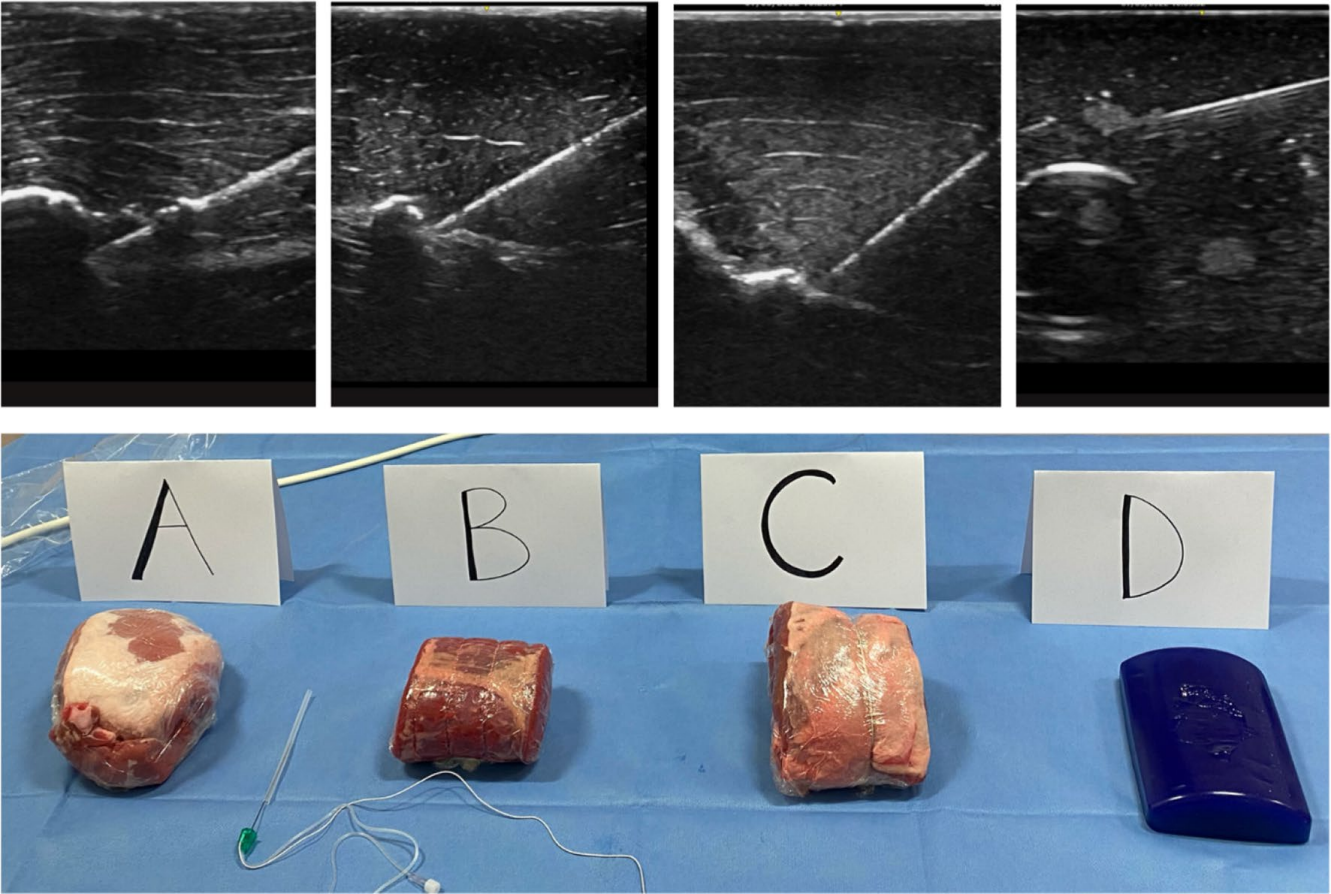
Specimens A–D with associated ultrasound pictures obtained

The primary outcome measure was to estimate subjectively which meat or phantom model best simulated the real patient by utilising a visual analogue scale (VAS) ranging from 0 to 10 (0 for least like human tissue and 10 for most like human tissue). All participants were asked to rank the realism of the images obtained (see Fig. 2) and the realism of the needling process for each specimen. The secondary outcome measure was to ascertain whether the experience or the total number of blocks previously performed by the participant influenced their subjective scorings of realism.

Mean specimen scores were analysed using repeated-measures analysis of variance, adjusted for years of anaesthetic experience and number of blocks previously performed, with between-specimen comparisons adjusted using the Bonferroni method. Analyses were done using Minitab (version 18) at a 5% significance level.

## Results

Thirty-eight participants were recruited. The number of peripheral blocks previously performed by the participants varied as described in Table 1.

**Table 1:** Number of blocks previously performed by participants

Number of blocks performed	Number of participants (%)
Less than 10	2 (5)
10–20	7 (19)
20–50	16(42)
More than 50	13 (34)

**Fig. 3:**
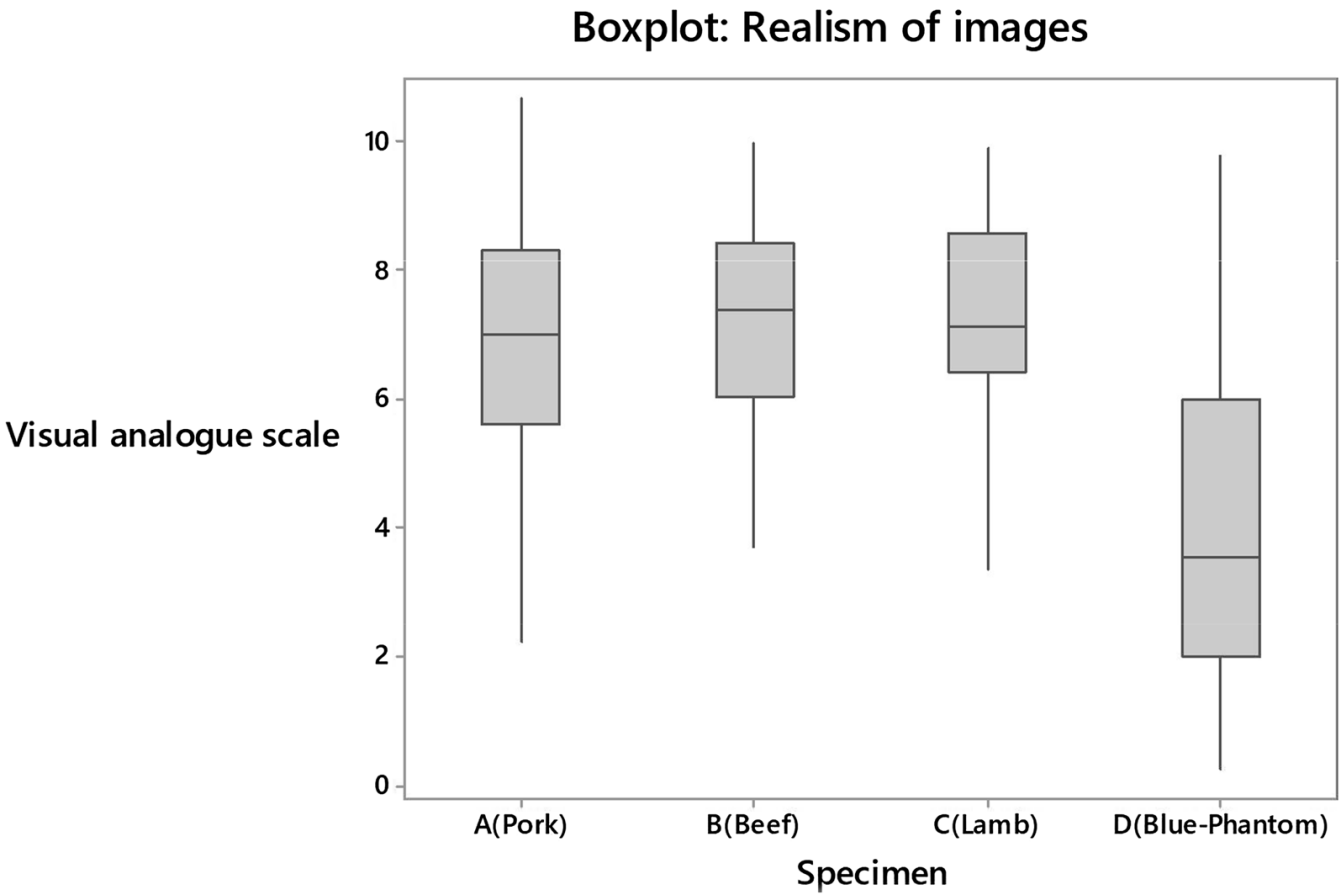
Boxplot comparing the realism of the appearances of ultrasound images using a visual analogue scale (0 least like human tissue–10 most like human tissue)

**Fig. 4:**
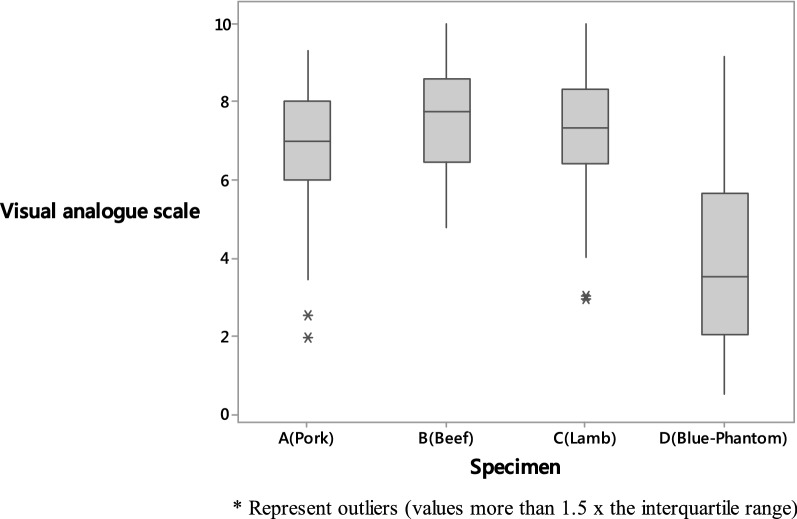
Boxplot comparing the realism of the ‘feel’ of needling using a visual analogue scale (0 least like human tissue–10 most like human tissue)

Realism was ranked according to the appearance of the ultrasound images, and ‘feel’ of the needling process as per Figs. 3, 4 and Table 2.

**Table 2:** Mean realism scores for the ‘look’ and ‘feel’ of each specimen

	A (pork) Mean (SD)	B (beef) Mean (SD)	C (lamb) Mean (SD)	D (blue phantom) Mean (SD)	*P* value (ANOVA)
Look	6.81 (2.04)	7.21 (1.66)	7.33 (1.74)	4.08 (2.35)	< 0.01
Feel	6.78 (1.70)	7.50 (1.41)	7.17 (1.76)	3.94 (2.27)	< 0.01

*SD* standard deviation, *ANOVA* analysis of variance

‘Look’ and ‘feel’ scores for specimen D were significantly lower than for specimens A, B and C (*p* = 0.01). There was no evidence of a difference in mean scores between A and B (*p* = 0.22), A and C (*p* = 0.55) or B and C (*p* = 1.00).

There was no significant difference in the ‘look’ or ‘feel’ assessments amongst those with different levels of experience (*p* = 0.62) or different numbers of blocks performed (*p* = 0.23).

## Discussion

These three types of meat were chosen to compare as they are easily accessible and offer slightly different properties. Beef brisket includes the superficial and deep pectoral muscles and a significant amount of connective tissue. The pork shoulder allowed comparison of a white cut of meat, with multiple muscles intertwined with fat. The thick layer of skin on the pork shoulder was removed as it reduced the ultrasonic windows. The lamb shoulder had a bone within it, creating signal dropout associated with bone on ultrasound. The results have shown that all the meats performed significantly better in realism of images and needling as compared to the blue phantom. There was no significant difference amongst the different types of meat, however the pork shoulder cost significantly less (pork shoulder £3.50/kg, beef brisket £7.49/kg, lamb shoulder £8.50/kg at the time of purchase).

The ideal phantom has been described to have tissue imaging similar to human tissue, be readily available, and be inexpensive. It should also have the ability to be used numerous times, hold a needle in place without generating too many needle tracks, and provide tactile feedback [11, 18]. Meat contains different fascial planes mimicking human muscle echogenicity and offers a realistic feel on needle advancement. Multiple needle passes in the specimens by candidates did not impair the integrity of the ultrasonic images throughout the day. It is reasonably inexpensive, easy to obtain, and relatively straightforward to prepare for use as a simulator (10 min preparation time per specimen). The main disadvantages are that meat phantoms require refrigeration, have a short lifespan, and are at risk of bacterial overgrowth. This was circumvented by only using each specimen for one day and then disposing of it thereafter. All the meats were stored in a cool bag in between testing sessions. The opportunity to perform full hand hygiene was provided and personal protective equipment was used by all participants. We ensured probe covers were used to protect the ultrasound machines and participants. The candidates did not experience any foul or strong smells despite not soaking the meat in alcohol as recommended by Xu et al. [19]. The length of time these phantoms would remain viable would depend on how they are stored between use, the freshness of the meat, and environmental conditions including temperature and humidity.

One limitation in this study was having multiple candidates scan and instrument the same specimens. As such, we did not allow participants to inject fluids into the specimens as this would have potentially distorted the ultrasonic images for subsequent participants [15]. Nevertheless, we anticipate that the meats could be used to teach the appreciation of local anaesthetic spread and for catheter insertion training which is another benefit over other types of model. This may lead to quicker degradation of the specimen, but, it is a viable option for low-budget training workshops. The catheter insertion capabilities of these meats would require further study.

Despite attempting to blind the meats with cling film, it was not possible to completely anonymise the identity of the different meats. Participants may have been able to decipher the different types of meats being scanned to a degree. This may have contributed to some bias in subjective scoring of realism. We can only speculate that this was reflected in the minority of participants and more time was spent on the scanning process rather than trying to decipher the identities of the specimens.

Another limitation that we forethought, was that variable degrees of experience may have influenced participants perception of the realism of the different specimens. However, it was statistically proven that the differing degrees of experience in performing blocks did not alter the results. This may be due to a specific endpoint of the ultrasonic images and the specific feel that all candidates were basing their VAS scores on.

## Conclusions

This study has shown that beef, pork and lamb joints are effective phantom models to use for simulation training for needling skills in UGRA. They are accessible, inexpensive, realistic, easily attainable, and do not have ethical approval or legal limitations to their use. We hope the use of meat models will make simulation practice easier to organise, thus making UGRA training more effective and accessible to anaesthetists of all levels and sonographers in other specialties.

## Data Availability

The datasets used and/or analysed during the current study are available from the corresponding author on reasonable request.

## Abbreviations

UGRA: Ultrasound-guided regional anaesthesia
VAS: Visual analogue scale
SD: Standard deviation
ANOVA: Analysis of variance
